# Autoantibody Diversity Is Augmented in Women with Breast Cancer and Is Related to the Stage of the Disease

**DOI:** 10.3390/curroncol30100634

**Published:** 2023-09-27

**Authors:** Jesús Pérez-Hernández, Rosalba León-Díaz, Alejandro Zentella, Edmundo Lamoyi, Marcela Esquivel-Velázquez, Antonia Barranca-Enríquez, Tania Romo-González

**Affiliations:** 1 Unidad de Medicina Experimental, Facultad de Medicina, Universidad Nacional Autónoma de México, Hospital General de México “Dr. Eduardo Liceaga”, Mexico City 06720, ZP, Mexico; jespertex@yahoo.com.mx; 2 Área de Biología y Salud Integral, Instituto de Investigaciones Biológicas, Universidad Veracruzana, Xalapa 91190, ZP, Mexico; rosleon@uv.mx; 3 Departamento de Medicina Genómica y Toxicología Ambiental, Instituto de Investigaciones Biomédicas, Universidad Nacional Autónoma de México (UNAM), Mexico City 04510, ZP, Mexico; azentell@iibiomedicas.unam.mx; 4 Departamento de Inmunología, Instituto de Investigaciones Biomédicas, Universidad Nacional Autónoma de México, Mexico City 04510, ZP, Mexico; lamoyi@unam.mx; 5 Laboratorio de Proteómica, Dirección de Investigación, Hospital General de México “Dr. Eduardo Liceaga”, Mexico City 06720, ZP, Mexico; 6 Centro de Estudios y Servicios en Salud, Universidad Veracruzana, Veracruz 91700, ZP, Mexico; abarranca@uv.mx

**Keywords:** breast cancer progression, serological data, diversity analysis, Mexican women, autoantibodies

## Abstract

Breast cancer (BC) is the most frequent malignant neoplasia and leading cause of cancer mortality for women. A timely diagnosis of BC is crucial to ensure the best chances of survival. Among the various screening tools for BC, antibodies directed towards self-antigens or tumor-associated antigens (autoantibodies) have emerged as an alternative to image-based screening modalities. However, little attention has been paid to the global diversity of autoantibodies. This work aimed to analyze the diversity of autoantibodies reactive to antigens expressed by the BC cell line T47D in the sera of Mexican women with BC, benign breast pathology (BBP), or without breast pathology (WBP). We found that the diversity of antibodies in the sera was higher in the BC and BBP groups than in the WBP group. Likewise, the diversity changed with the progression of BC. Our results show and measure the complexity of the antibody response in breast health and disease.

## 1. Introduction

Breast cancer (BC) is the most common malignancy in women worldwide and is the leading cause of cancer mortality [1]. In Mexico, BC is the leading cause of cancer mortality in women, with an incidence of 18.55 per 100,000 and mortality of 17.94 per 100,000, with ductal breast carcinoma being the most common type [2]. Late diagnosis impacts the survival rate; in Mexico, most cases of BC are diagnosed late, which places the 5-year survival rate at 36%. Given the relatively high morbidity and mortality rates associated with BC, public health systems around the world have implemented programs to increase the life expectancy of women, based on timely diagnosis after periodic physical examination of the breasts, image-based screening, and identification of blood molecular markers. Unfortunately, this strategy has been insufficient in decreasing BC morbidity and mortality, especially in countries with developing economies, where population-wide medical mentoring and education is deficient, and the population has limited access to screening programs and treatments [3].

Autoantibodies have been studied as candidates for use as biomarkers or tools for the early detection of cancer, due to their ability to recognize antigens produced by the body’s cells in low concentrations in a constitutive manner. In cancerous processes, tumor-associated antigens arise through the aberrant expression of normal and mutated proteins, which may also show post-transcriptional and post-translational alterations and abnormal cellular localization [4]. BC tumors are very heterogeneous in their intrinsic (molecular) and extrinsic (microenvironment) characteristics as they follow a progression process. With this in mind, it is unsurprising that there may be a large diversity of aberrantly expressed proteins, which could affect the composition of circulating antibodies [5].

Several proteins detected by autoantibodies in women with BC have been candidates for possible biomarkers, i.e., p53, c-myc, HER-2, NY-ESO-1, BRCA1, BRCA2, and MUC1, among others [4,6,7]. However, insufficient sensitivity and/or specificity and often contradictory results are found for the same antibody in different studies [7]. In Mexico, there are few reports on the correlation between these molecules with prognosis. Only in 2012 and 2015, two tumor-associated antigens were reported: alpha 1-antitrypsin and alpha 2HS-glycoprotein, respectively [8,9]. More recently, the presence of full-length recombinant proteins—alpha 1-antitrypsin (A1AT), triose-phosphate isomerase 1 (TPI1), peptidyl-prolyl cis-trans isomerase A (PPIA), and peroxiredoxin 2 (PRDX2)—as microarrays evaluated potential antigens associated with early-stage breast cancer tumors [10].

These works show the scarce efforts to learn more about the potential of autoantibodies as adjuvant tools for diagnosis, not to mention the diversity of autoantibodies in the different stages of the disease, the existing reports of the distinguishable differences in serum IgG profiles between BC patients and healthy ones [5], or as in the case of autoimmune diseases, where differences in the diversity of autoantibodies are observed according to the progression of the disease [11]. 

Therefore, in this study, applying population biology tools, we analyzed the diversity of antibodies reactive to antigens expressed in ductal BC-derived T47D cells with Western blot. We included serum samples from Mexican women with breast cancer (BC) at different stages of the disease, benign breast pathology (BBP), or without breast pathology (WBP). 

## 2. Materials and Methods

### 2.1. The Study Design

We conducted an observational cross-sectional study in Mexico City. It met the STROBE criteria, as recommended [12].

The study was conducted at the Hospital General de México Dr. Eduardo Liceaga and the Biomedical Research Institute (Universidad Nacional Autónoma de México-UNAM, Mexico City, Mexico). The study followed the principles of the Declaration of Helsinki. It was revised and approved by the Ethics and Research Committees of the Hospital General de México Dr. Eduardo Liceaga (Approval No: DI/12/111/03/064).

### 2.2. Participants

Mexican women aged between 16 and 79, with at least two generations born in Mexico, were invited to participate through an open call at the Biomedical Research Institute (UNAM, Mexico) or were invited to participate at their first gynecologic-oncological appointment (before any previous cancer treatment) at the Hospital General de México “Dr. Eduardo Liceaga”. Women who accepted the invitation to participate in the study signed the informed consent after a thorough explanation and were enrolled. Enrolled patients were classified into three groups according to their clinical evaluation: breast cancer (BC), benign breast pathology (BBP), and without breast pathology (WBP).

### 2.3. Sample Size

Since this was an exploratory study, the sample size was 50 subjects per group. A convenience sampling method was used.

### 2.4. Blood Samples

Ten milliliters of venous blood was withdrawn from each woman using sterile, disposable BD Vacutainer^®^ kits. Blood samples were left to clot at room temperature and then were centrifuged at 1500 rpm in a clinical centrifuge (Eppendorf) at room temperature for 5 min. The serum collected was aliquoted (500 µL per sample) and stored at −80 °C until used. 

### 2.5. T47D Cell Line Culture

The breast cancer cell line T47D (ATCC; Manassas, VA, USA) was cultured in phenol-free RPMI 1640 medium, supplemented with 10% fetal calf serum, 100 U/mL penicillin, 100 mU/mL streptomycin, and 250 ng/mL amphotericin B on plastic culture plates (Costar, Cambridge, UK) under 95% humidity and 5% CO_2_ at 37 °C. Cells with 85 to 90% confluence were harvested using cold Versene (PBS + EDTA 0.02%) and pelleted via centrifugation at 1500 rpm for 5 min at room temperature. Cell pellets were washed three times with PBS and stored at −80 °C. 

### 2.6. T47D Protein Extraction

Cultured T47D cells were used as the source of antigens for immunoblots. Cell pellets were lysed in a buffer containing 4% (*w/v*) CHAPS (Bio-Rad, Hercules, CA, USA), 7 M urea (SIGMA-Aldrich, San Luis, MO, USA), 65 mM DTT (Promega, Madison, WI, USA), and a protease inhibitor cocktail (10 μL per 875 μL of lysis buffer; Halt, PIERCE) through 5 freeze–thaw cycles. Lysates were centrifuged at 16,000× *g* for 10 min at 4 °C; supernatants were recovered, pooled, aliquoted, and stored at −80 °C until further use. Protein concentration was determined using the Bradford assay (Bio-Rad, Hercules, CA, USA).

### 2.7. SDS-PAGE and Western Blot

Protein extracts from T47D cells (100 µg) were electrophoresed through polyacrylamide gels (4–20% TGX, Bio-Rad Laboratories, Hercules, CA, USA) at 80 V for 2 h. They were then transferred onto nitrocellulose membranes (High Bond, Amersham Biosciences, Amersham, UK) at 100 V for 1.25 h using a mini Trans-Blot cell (Bio-Rad, Hercules, CA, USA). Protein transfer was assessed by staining the membranes with copper phthalocyanine tetrasulphonic acid (Sigma-Aldrich) diluted in 12 mM HCl; membranes were scanned and then de-stained. Each membrane’s upper and lower boundaries were lined with a pencil, and seventeen or eighteen four-millimeter-wide strips were cut vertically between the borders. Membranes were then blocked with 5% (*w/v*) skimmed milk (Svelty, Nestlé, Lagos de Moreno, Jalisco, México) diluted in PBS with 0.3% Tween 20 (PBS-T) for 16 h at 4 °C. Strips were individually incubated with a different serum diluted 1:300 in 2 mL of PBS-T during 5 h at room temperature. The serum coded as 111 was used as an internal control for matching bands between membranes. After a thorough wash with PBS-T, the bound antibodies were detected by incubating the membrane strips with goat anti-human IgG H + L-HRP antibody (1:2500; Thermo Scientific, Waltham, MA, USA) for one hour at room temperature. Peroxidase activity was revealed by incubating the membranes with 0.1 mg/mL 3, 3′-diaminobenzidine tetrahydrochloride (Sigma-Aldrich, San Luis, MO, USA) and 0.015% hydrogen peroxide in PBS-T for 5 min at room temperature. The peroxidase reaction was stopped by gently washing the strips five times with deionized water, and the strips were left to air dry.

### 2.8. Image Analysis

Strips were digitized (Hewlett-Packard Scanjet G4050, Böblingen, Germany) in TIF format at a resolution of 300 dpi; brightness, contrast, and gain were kept constant in all image acquisitions. Once captured, strips were aligned in Adobe Photoshop CS5 using the pencil-drawn lines on the membrane as a reference. Bands were detected using Quantity One Software 4.6.3 (Bio-Rad, Hercules, CA, USA), and banding patterns in stripes from different membranes were compared using the banding pattern obtained in the control strips (serum 111) (Supplementary Material, Figure S1). 

A database was constructed with the data from each band’s presence (1) or absence (0), numbering the bands as their molecular weight decreased. 

### 2.9. Statistical Analysis 

Means, variances, and standard deviations were calculated. Normality was assessed using the Kolmogorov–Smirnov test. Levene’s test was used to determine the homogeneity of variances. Groups were compared using one-way ANOVA, Kruskal–Wallis, or Chi-squared according to the type of variable and its distribution. Odds ratios were calculated for each band by dividing its frequency in one group by its frequency in another. Venn diagrams were constructed using an online program (http://bioinformatics.psb.ugent.be/beg/tools/venn-diagrams, accesed on 27 July 2022). Dice similarity coefficients were calculated, and similarity, phenograms were made using the Mega 6.0 software [13] and the Neighbor-Joining clustering method. Phenogram branches were colored using Photoshop CS5. 

The Shannon diversity H index (was calculated for each group as follows: $H=-\sum_{j=1}^{S}p_{i}(ln(p_{i})$; where S is the total number of bands in each group and $p_{i}$ is the proportion of S of the *i* band. To compare the diversity between the groups, the effective number of species ($ENS=e^{H}$) was calculated. The H index was compared between groups using Hutcheson’s *t*-test.

## 3. Results

### 3.1. Characteristics of Participants

There were no differences in weight, height, BMI, number of abortions, or total breastfeeding length in months (after adjusting for the number of births). However, women in the BC group were older (*p* < 0.001) and had more gestations and births by vaginal delivery than women with BBP and WPB (*p* < 0.001, after adjustment for age). Likewise, women with BC and BBP had higher abdominal and hip perimeters than women WBP (*p* = 0.003 and *p* = 0.006, respectively). Worldwide, age is considered a breast cancer risk factor [14], while the length of breastfeeding is regarded as a protective factor [15]. However, in our sample, breastfeeding time was higher in the BC group (51.28 ± 13.45 in BC vs. 39.68 ± 12.33 in WBP and 40.42 ± 11.69 in BBP groups), as well as the number of gestations (Table 1).

In the BC group, the most frequent type of breast carcinoma was invasive ductal carcinoma (35 patients, 70%) (Table 2). Equally less frequent types were invasive lobular carcinoma, tubular ductal carcinoma, mucinous carcinoma, and tubulo-lobular carcinoma, which only accounted for one patient each. More than half of the women were in stages II and III (34 patients, 68%), while only 6 (12%) and 5 (10%) were in stages I and IV, respectively (Table 2). In Mexico, as in the rest of the world, breast ductal carcinoma is the most common histological type of BC, constituting 68% of all breast tumors [16]; our sample is consistent with these data. No differences were found between women with BC at different stages of the disease in age, BMI, pregnancies, vaginal or C-section deliveries, or breastfeeding length.

### 3.2. Autoantibodies from BC Patients Recognize a Larger Number of T47D Antigens vs. BBP and WBP

**Figure 1:**
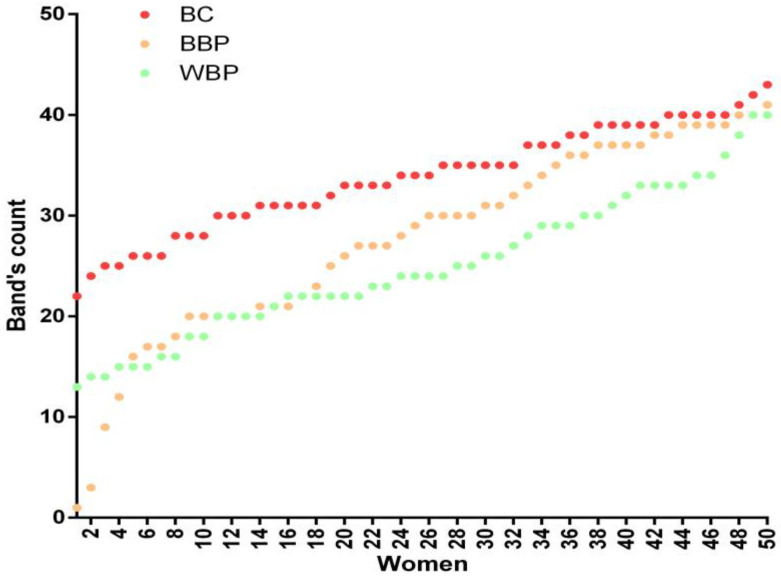
Number of bands recognized by the sera of each of the 150 patients (50 patients per group) of the three groups: BC (breast cancer), BBP (benign breast pathology), and WBP (without breast pathology).

Antibodies in the sera detected numerous protein bands of different molecular weights in all groups (228 bands, Supplementary Material, Table S1). Adding the total number of bands perceived per individual serum in each group, sera from BC patients observed more bands (Σ = 1687) than those from BBP (Σ = 1379) or WBP (Σ = 1250). Also, BC patient sera recognized the highest percentage of the 228 different bands (79.8%), whereas BBP patient sera recognized 77.6% and WBP sera recognized 75%. Sera from individual women in the BC group showed the highest number of bands detected (Figure 1).

### 3.3. Sera Antibody Patterns of BC and BBP Groups Are Very Similar

**Figure 2:**
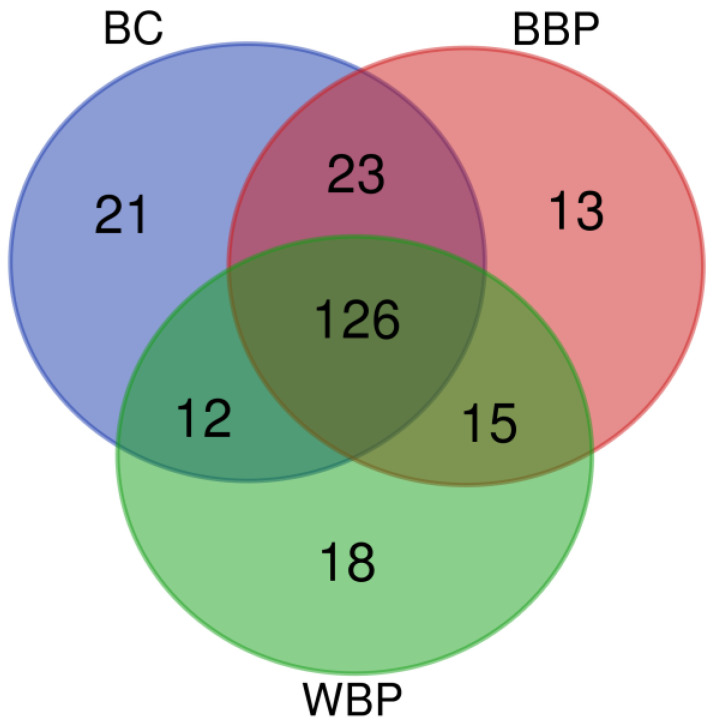
Venn diagram showing the number of bands recognized by sera from patients from each of the three groups and the intersections. BC (breast cancer), BBP (benign breast pathology), and WBP (without breast pathology).

To identify whether there were unique characteristics for any of the groups, we constructed Venn diagrams in which each band is represented according to its recognition by the sera of women from the different groups (Figure 2). Twenty-one bands were detected exclusively by sera from women in the BC group, while thirteen were unique to the BBP group and eighteen to the WBP group. Of the remaining 176 bands, 43 were distributed in the intersections: BC∩BBP (23 bands), BC∩WBP (12 bands), and BBP∩WBP (14 bands).

**Figure 3:**
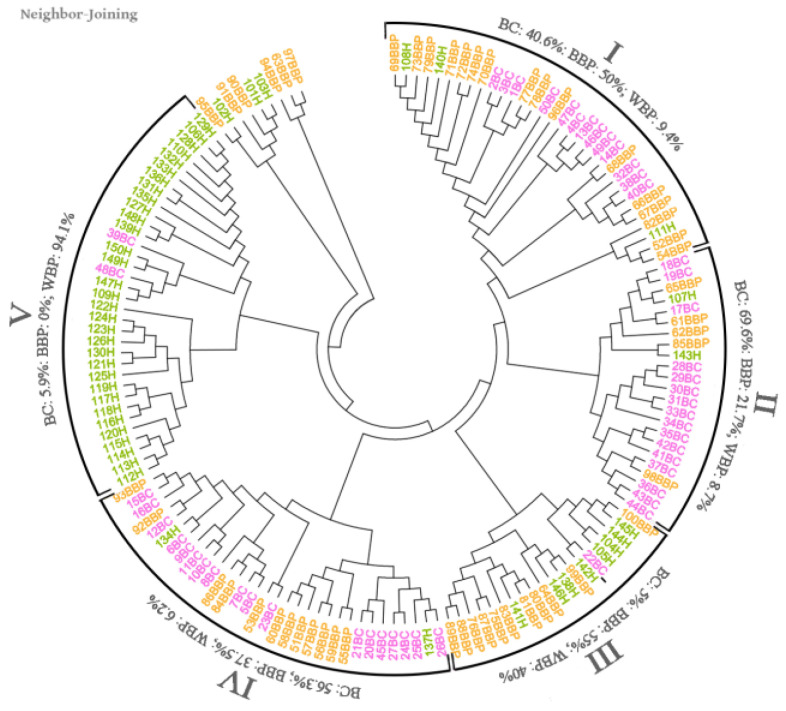
Similarity phenogram constructed based on Dice similarity coefficients. Five principal branches can be distinguished; each preferentially concentrates patients from specific groups. BC (breast cancer), BBP (benign breast pathology), and H = WBP (without breast pathology).

Very different banding patterns were obtained for each serum; to identify whether these patterns could group the sera according to their health status, we constructed a phenogram (Figure 3). The phenogram revealed the existence of five branching data clusters. Clusters I, II, and IV comprise mainly patients with BC and BBP, while cluster III encompasses mainly patients without breast cancer (BBP and WBP). On the other hand, cluster V consists mainly of patients without breast pathology, either benign or malignant.

### 3.4. Diversity in the Antibodies Repertoire Is Increased in Patients with BBP and BC

To explore the diversity of antibodies capable of recognizing antigens from the T47D cell line, we calculated the effective number of species (ENS) as an indicator of such diversity, which considered the best indicator of diversity [17].

**Figure 4:**
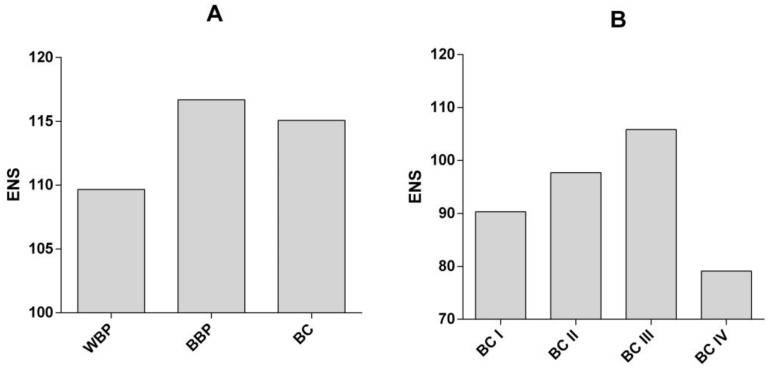
Antibody diversity (ENS) in (**A**) WBC, BBP, and BC groups, and (**B**) in different stages of BC. BC (breast cancer), BBP (benign breast pathology), and WBP (without breast pathology).

The ENS was lower for the WBP group than the BBP and BC groups (Figure 4A), reflecting a greater diversity of antibodies present in the BBP and BC groups, both of which display breast pathology, whether benign or malignant. We then calculated the ENS considering the stage of BC (I to IV). The ENS increased from stages I to III and drastically dropped in stage IV BC patients (Figure 4B), demonstrating a lower diversity of antibodies in this stage compared to the previous stages. As with the number of bands, the drop in the diversity of antibodies at this stage of BC is probably a reflection of the immunosuppression phenomena observed as the BC progresses [18]. These differences in diversity are statistically significant, as can be observed in Figure S2 (Supplementary Material) of the Shannon diversity index.

## 4. Discussion

Breast cancer continues to be the leading cause of cancer death in women in the world, mainly in emerging economies such as Mexico. In this regard, various authors have analyzed and measured the presence of autoantibodies in various types of cancer (including breast cancer), and some have even evaluated the effectiveness for the recognition of breast cancer antigens [3,4]. Even though their concentration and variety differ significantly among subjects, the fact that autoantibodies’ individual patterns remain relatively stable throughout life but shift when the individuals begin developing a disease or if they become ill led us to think that they may be used as biomarkers [4,5]. In this sense, here we explore the diversity of antibodies reactive to antigens expressed in ductal BC-derived T47D cells with Western blot, from Mexican women serum samples with BC at different stages of the disease, BBP, or WBP.

On the other hand, the number of total bands detected using the sera from BC patients increased. The sera of patients with BC recognized a larger number of bands than the sera from BBP and WBP patients (Figure 1). These differences are most likely due to the immunological response raised by the tumor’s presence and the variety of aberrantly expressed proteins [5], which is reflected in a greater variety of antibodies. BC and BBP groups share the largest number of bands in the Venn diagram (Figure 2), aside from the bands shared by the three groups, while the bands shared with the WBP group were lower in both cases. In addition, our analysis showed that twenty-one bands were recognized solely by the sera of patients with BC. Although those 21 bands can point to specific antigens to be used as biomarkers, the low frequency of recognition (<10% of BC patients) makes them unsuitable for this purpose. Nonetheless, it is important to note that, within the different banding patterns obtained, the similitude analysis depicted in the phenogram (Figure 3) revealed the existence of well-defined branches that preferentially group the patients according to their pathology (WBP, BBP, or BC). Likewise, the clusters we found in the phenogram show that, in most cases, the banding patterns of BBP women are more similar to those of BC women, except in one of the branches, where they clustered with WBP women. BBP is frequently found in women and involves several changes in the breast tissue, some similar to those occurring in BC [19], which may explain the grouping behavior observed. Likewise, it has been reported that sera IgG antibody profiles differ between BC and healthy patients using protein microarrays [5], which were the groups most clearly differentiated in the phenogram. Therefore, the follow-up of BBP patients would have been necessary to investigate whether the women with BBP who had been grouped with BC could be related to the risk of future BC development.

Given that the variability of autoantibodies is of medical interest for diagnostic purposes and their participation in both the pathogenesis and progression of cancer, including breast cancer, is widely documented [20], here we explore the diversity of antibodies reactive to antigens expressed in ductal BC-derived T47D cells with Western blot, from Mexican women serum samples with BC at different stages of the disease, BBP, or WBP. For this, a powerful ecology tool was used, which is the statistical calculation of diversity through the effective number of species (ENS). ENS refers to the number of equally abundant species needed to obtain the same mean proportional species abundance observed in the dataset of interest (where all species may not be equally abundant) [17]. The ENS is calculated using the Shannon index; it allows for the measurement of diversity in the unit “number of species” rather than a non-linear, dimensionless index. For this, ENS is considered the best indicator of diversity, although most statistical tools have been proven for H index when comparing between communities. For this reason, we statistically compared the H Index, which follows the same behavior between the groups in the reported graphs. We found that the recognition of epitopes derived from T47D cells detected in the form of bands in the immunoblot by the sera of patients with BC was significantly larger than that of the BBP and WBP groups. Autoantibodies are immunoglobulins that recognize self-antigens. It is now accepted that they form part of the normal protein components of various body fluids, including the blood serum [4]. Until a few years ago, it was considered that their production was mediated solely by immune cells; however, in recent years, the active participation of autoantibodies in the carcinogenesis process of different types of tumors, without excluding BC, has been demonstrated [18]. 

For the diversity assessment, our data reveal an increase in the ENS of autoantibodies in BBP and BC groups, compared to the WBP group. Furthermore, the number of total bands detected by sera from BC patients increased according to the stage of the disease, from stage I to III (Σ = 209, Σ = 391, and Σ = 776, respectively). In contrast, a drastic decrease in band recognition was observed in stage IV (Σ = 162). Here, we hypothesized that this phenomenon may not only reflect the changes in the tumor’s aberrantly expressed proteins as the disease progresses, but also demonstrate the immunosuppression that occurs at later stages [18], or it could reflect the immunological processes associated with changes in the breast tissue, either malignant or benign, in which we considered that the antibody response focuses on a broader set of antigens probably related to those changes, which are not present or are present to a lesser extent in the WBP patients. This hypothesis is supported by the fact that an increase in B cell count has also been linked to a better prognosis for breast cancer [20], but they can also contribute to tumor progression [21] and breast cancer metastasis. B cells contribute to an increase in circulating immune complexes (CICs), created when antibodies bind to floating antigens [22]. These complexes can accumulate in tissues, induce the formation of sites of inflammation, activate complement pathways, and interact with Fc-gamma receptors (FcγR) on the surface of leukocytes [23]. These elements can cause chronic inflammation by activating myeloid cells with the participation of the Fc receptor. Therefore, it is not surprising that high CIC levels are associated with a poor prognosis in some patients diagnosed with, for example, breast, genitourinary, head, and neck cancers [24,25]. On the other hand, B cells have cancer-regressive activity, mainly through antibody production [20], which can reduce early neoplasms [26]. In this sense, our finding of an increase in the ENS from stages I to III of BC and the drastic reduction in the ENS in stage IV agrees with this reported role of B cells and antibodies as breast cancer progresses. Stage IV breast cancer is characterized mainly by a metastatic state, in which the immune response is inefficient in eliminating cancer cells. In this regard, Székely et al. [27] have reported that the number of tumor-infiltrating lymphocytes (TILs) and the protein expression of programmed death ligand 1 (PD-L1) are substantially lower in samples obtained from metastases derived from patients diagnosed with breast cancer than in samples obtained from patients diagnosed with primary tumors, as well as a significantly lower expression of CD27 and its ligands CD70, CD29, and CD40L, which are necessary for T cell activation. In addition, the expression of activated T cell transcription factors NFAT-1,-2, as well as granzyme, granulysin, IFNγ, and interferon-regulated genes (STAT1, IRF-1,-4,-7, IFI-27,-35, MX1) was also lower, which is consistent with an inactive state of T lymphocytes in metastases. 

The variability of autoantibodies is of medical interest since they participate in both the pathogenesis and progression of breast cancer [22]. In recent years, tumor-infiltrating B cells (TIL-B) have been associated with a better prognosis in breast cancer patients, within which virgin B cells, memory B cells, and plasma cells have been described [28,29]. However, the presence of intratumoral IL10+ Bregs is a poor prognostic feature in breast cancer. This enrichment of Bregs was attributed to infiltration and regulation by CD33+ myeloid-derived suppressor cells (MDSCs) that contributed to an immunosuppressive tumor microenvironment [30,31], the latter exhibiting the most significant frequency of somatic IgH hypermutation compared to other TIL-B subsets, with most clones sharing identical IgH sequences [29], and suggesting a high specificity of Ig against tumor antigens. However, the rate of somatic hypermutation of plasma cell IgH is downregulated in primary breast tumors compared to those present in peripheral blood mononuclear cell (PBMC) samples, indicating the potential ability of tumor cells to suppress the local humoral response [29], which is reflected in a significant decrease in the diversity of autoantibodies, although not necessarily impacting the number of autoantibodies, which becomes evident in late-stage disease.

Finally, in this article, we present for the first time a study on the diversity and abundance of autoantibodies for breast cancer in the Mexican population. Although the sample is small for each group, the inclusion of patients before the initiation of cancer treatment and the selection criteria allowed us to compare patients in similar conditions. Likewise, we used community ecology tools to compare the different groups; to our knowledge, this is the first time such tools have been applied to the analysis of the diversity of antibodies in breast cancer research. This type of research has the potential to provide more information about different possible scenarios in breast cancer, from subtypes of breast cancer to the monitoring of the progression and response to treatments. In this sense, our study provides a research strategy to be applied in the abovementioned scenarios of breast cancer as well as in other types of cancer or even in autoimmune diseases. Despite the above, this type of work still needs to be more widely disseminated among the clinical community in order to (1) favor research with a diversity of samples (biopsies and serum) and patients with different stages and treatments, and (2) propose the use of autoantibodies as diagnostic tools, in addition to the immunohistochemical analyses that are already performed to identify the tumor’s hormonal susceptibility.

## 5. Conclusions

The IgG antibody diversity, assessed using the ENS, is higher in BC and BBP sera than in the sera of women WBP. The diversity increased from stages I to III of breast cancer and was drastically diminished in stage IV. This diversity could be a reflection of the immunological recognition of changes in the breast tissue, as well as the immunosuppression that accompanies metastatic cancer. More research is necessary to fully understand the mechanisms behind this phenomenon and to determine the specific antigens that contribute to the diversity.

## Figures and Tables

**Table 1:** Characteristics of women included in the study.

	WBP	BBP	BC	*p*
Age, years	39.68 ± 12.33	40.42 ± 11.69	51.28 ± 13.45	<0.001 ^a^
Weight, kg	61.75 ± 9.15	65.20 ± 13.56	66.28 ± 15.70	0.362 ^b^
Height, m	1.56 ± 0.06	1.54 ± 0.08	1.54 ± 0.08	0.335 ^a^
BMI, kg/m^2^	25.51 ± 3.77	27.56 ± 5.46	27.82 ± 5.30	0.108 ^b^
Gestations, no.	1.36 ± 1.54	2.11 ± 1.82	3.48 ± 2.05	<0.001 ^b^
Mode of births				
Vaginal	0.78 ± 1.26	0.95 ±1.29	2.56 ± 2.16	<0.001 ^b^
C-section	0.34± 0.75	0.66 ± 1.02	0.54 ± 0.93	0.243 ^b^
Abortions, no.	0.17 ± 0.50	0.37 ± 0.69	0.38± 0.98	0.276 ^b^
Total breastfeeding length, months	3.89 ± 6.84	8.88 ± 10.32	11.96 ± 9.95	<0.001 ^b^
Abdominal perimeter, cm	85.12 ± 17.92	93.34 ± 11.90	95.67 ± 13.33	0.003 ^b^
Hip perimeter, cm	91.90 ± 19.41	99.33 ± 12.32	102.16 ± 12.51	0.006 ^b^

Data are presented as mean ± standard deviation. BMI: Body Mass Index. ^a^ One-way ANOVA. ^b^ Kruskal–Wallis.

**Table 2:** Clinical characteristics of the BC patients in relation to the stage of the disease.

Stage	I (*n* = 6)	II (*n* = 11)	III (*n* = 23)	IV (*n* = 5)	Not Classified (*n* = 5)	*p*
Age, years	50 ± 20.09	51.45 ± 14.96	51.13 ± 12.39	50.6 ± 13.37	53 ± 12.94	0.999 ^a^
BMI, kg/m^2^	28.94 ± 8.6	27.09 ± 5.18	27.95 ± 5.01	28.62 ± 3.5	28.88 ± 5.24	0.944 ^a^
Gestations, no.	3.17 ± 1.17	3.64 ± 1.75	3.43 ± 2.57	3.4 ± 1.67	4 ± 1	0.983 ^a^
Mode of births						
Vaginal	2.64 ± 1.63	2.73 ± 2.33	2.22 ± 2.33	3 ± 2.24	3.67 ± 1.53	0.814 ^a^
C-section	0.33 ± 0.82	0.81 ± 1.25	0.57 ± 0.9	0.4 ± 0.89	0.25 ± 0.5	0.793 ^a^
Abortions, no.	0.17 ± 0.41	0.09 ±0.3	0.65 ± 1.34	0.2 ± 0.45	0 ± 0	0.473 ^a^
Total breastfeeding length, months	16 ± 10.81	10.82 ± 7.65	11.75 ± 12.46	10 ± 5.1	12.67 ± 1.15	0.865 ^a^
*Histological subtypes*						
Invasive ductal carcinoma, *n*(%)	3	8	16	5	3	0.993 ^c^
Invasive lobular carcinoma, *n*(%)	0	0	1	0	0	
Tubular ductal carcinoma, *n*(%)	0	0	1	0	0	
Mucinous carcinoma, *n*(%)	0	1	0	0	0	
Tubulo-lobular carcinoma, *n*(%)	0	0	1	0	0	
*Tumor molecular characteristics*						
SBR	8.67 ± 0.58	6.29 ± 1.5	7.64 ± 1.55	7.33 ± 1.53	4 ± 0	0.077 ^b^
ER	15 ± 21.21	81.11 ± 9.28	44.71 ± 39.86	12 ± 28.63	70 ± 34.64	0.009 ^b^
PR	20 ± 28.28	60 ± 37.8	22.35 ± 30.32	17 ± 38.01	40 ± 56.57	0.210 ^b^
Ner2/Neu	50 ± 70.71	10 ± 26.46	31.76 ± 44.61	40 ± 54.77	66.67 ± 57.74	0.456 ^b^
Ki67	27.5 ± 31.82	36.67 ± 28.83	51.47 ± 30.71	50 ± 23.43	26.67 ± 20.82	0.466 ^a^

Data are presented as mean ± standard deviation or frequency(percentage). SBR: Scarf–Bloom–Richardson. ER: estrogen receptor. PR: progesterone receptor. ^a^ One-way ANOVA. ^b^ Kruskal–Wallis. ^c^ Chi-squared.

## Data Availability

The data supporting the reported results can be found in Supplementary Material as Figure S1 (Western blots) and Table S1 (database).

## Supplementary Materials

The following supporting information can be downloaded at: https://www.mdpi.com/article/10.3390/curroncol30100634/s1. Figure S1. Crude images of the Wester Blots performed. Each strip corresponds to a different serum. The numbers are the sample IDs. The Serum 111 was used as an internal control in all membranes. C(-): Negative control, only secondary antibody (a Human IgG-HRP (H+L)). GAPDH: glyceraldehyde-3-phosphate dehydrogenase, revealed with a specific monoclonal antibody and with anti-mouse IgG-HRP (H+L) as the secondary antibody. Figure S2. Comparison of Shannon Diversity Index between groups. (A) The Shannon Diversity index (H) was calculated for each group (BC, BBP, and WBP). (B) H for each stage of BC. Error bars correspond to the confidence intervals. The Hutcheson t-test was used to compare between groups. Table S1. Database.
