# Supplementary material for: Autoantibody Diversity Is Augmented in Women with Breast Cancer and Is Related to the Stage of the Disease
Source: Curr Oncol. 2023 Sep 27;30(10):8793–804. doi: 10.3390/curroncol30100634 (PMC10605201; doi:10.3390/curroncol30100634)

Figure S1. Crude images of the Wester Blots performed. Each strip corresponds to a different serum. The numbers are the sample IDs. The Serum 111 was used as an internal control in all membranes. C(-): Negative control, only secondary antibody (α Human IgG-HRP (H+L)). GAPDH: glyceraldehyde-3-phosphate dehydrogenase, revealed with a specific monoclonal antibody and with anti-mouse IgG-HRP (H+L) as the secondary antibody.

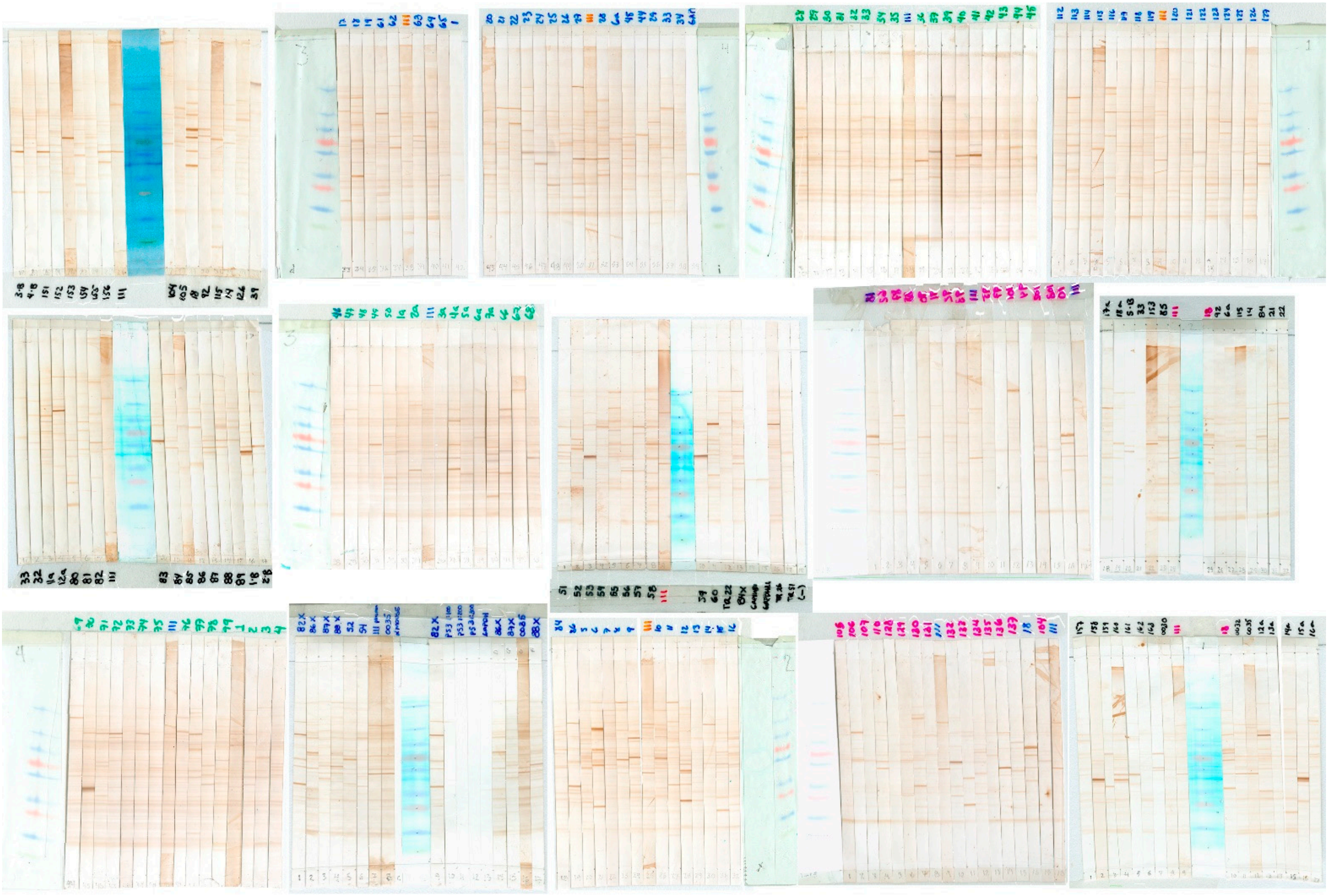

Figure S2. Comparison of Shannon Diversity Index between groups. A) The Shannon Diversity index (H) was calculated for each group (BC, BBP, and WBP). B) H for each stage of BC. Error bars correspond to the confidence intervals. The Hutcheson t-test was used to compare between groups.

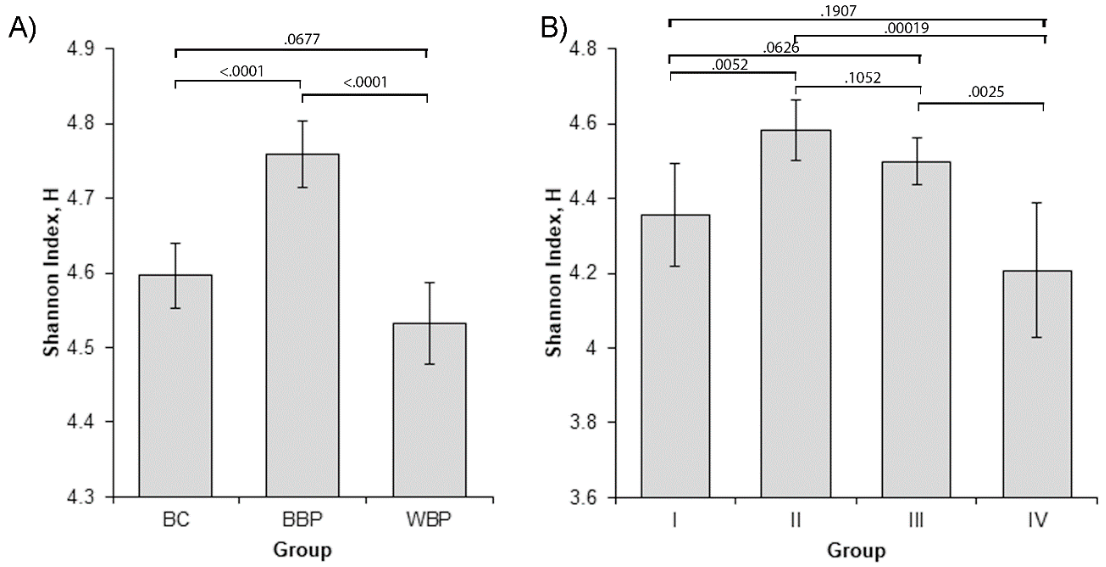

Supplement: Supplementary file 1 [file curroncol-30-00634-s001.zip › Supplemental figures S1 and S2.pdf]
